# Endovascular Therapy of Ruptured Aneurysms on Moyamoya Collateral Vessels: Two Cases

**DOI:** 10.3390/medicina60091499

**Published:** 2024-09-14

**Authors:** Pavel Ryška, Miroslav Lojík, Jiřina Habalová, Carmen Kajzrová, Tomáš Česák, Eva Vítková, Michael Bartoš, Zdeněk Bělobrádek, Antonín Krajina

**Affiliations:** 1 Department of Radiology, University Hospital, 50005 Hradec Kralove, Czech Republic; 2 Faculty of Medicine in Hradec Kralove, Charles University, 50003 Hradec Kralove, Czech Republic; 3 Department of Neurosurgery, University Hospital, Faculty of Medicine, Charles University, 50005 Hradec Kralove, Czech Republic; 4 Department of Neurology, University Hospital, Faculty of Medicine, Charles University, 50005 Hradec Kralove, Czech Republic

**Keywords:** moyamoya, acute stroke, endovascular therapy

## Abstract

*Background*: Using two case reports of adult women with moyamoya disease presenting with intracranial hemorrhage from ruptured aneurysms on moyamoya collateral vessels, we aim to demonstrate the potential for effective endovascular treatment navigated by CT angiography, digital subtraction angiography, and flat panel CT. *Case 1 Presentation*: A 45-year-old female patient with sudden onset of headache, followed by somnolency. CT scan showed a four-ventricle hematocephalus caused by a 27 × 31 × 17 mm hematoma located in the left basal ganglia. Angiography revealed a 3 mm aneurysm on hypertrophic lenticulostriate artery bridging the M1 occlusion. Selective catheterization and distal embolisation with acrylic glue was done. *Case 2 Presentation*: A 47-year-old woman was admitted for a sudden onset of severe headache, CT scan showed four-ventricle hematocephalus. A 4 mm aneurysm on the collateral vessel–anterior chorioidal artery bridging the closure of the terminal segment of the internal carotid artery was diagnosed as the source of bleeding. Selective catheterization and distal embolisation with acrylic glue was done. *Conclusions*: Selective embolisation of ruptured aneurysms on moya moya collaterals is a simple, effective, and safe procedure when relevant microcatheters are used with imaging software navigation such as 3D DSA, 3D road map and flat-panel CT.

## 1. Introduction

Chronic progressive non-inflammatory and non-atherosclerotic stenosis of the supraclinoid segments of both internal carotid arteries was first described in Japan; however, in the last decades of the 20th century, this disease was predominantly diagnosed in women of the global population [1,2]. The name of the disease, Moyamoya disease (MMD) is derived from the angiographic image of typical collateralization by multiplication and hypertrophy of the perforating cerebral arteries forming the image of a “puff of cigarette smoke.” Less commonly, one side and also the posterior circulation may be affected [3]. In contrast to MMD, a similar pattern of collaterals may be seen in associated diseases including genetic diseases (Trisomy 21, sickle cell anemia, pial arteriovenous malformation, and others). Unrecognized hematologic diseases may be another rare cause of intracranial hemorrhage in young individuals [4].

When a patient has similar vasculopathy in the setting of another disease then it is known as Moyamoya syndrome [5]. For MMD, we use a classification based on the degree of progression followed by progressive collateral loss with disease progression. A more recent classification is based on MRI findings of ischemia, including watershed infarcts, hemorrhages, and cerebrovascular reserve capacity, in addition to angiographic findings [6].

The most common clinical symptoms of ruptured collateral aneurysms in MMD are headache with or without nausea (in 36% of patients), impaired consciousness (in 24% of patients), focal neurological deficit (in 7% of patients), and a combination of focal neurological deficit with impaired consciousness (in 8% of patients) [7]. Conservative treatment carries a 40% risk of recurrent hemorrhage within the first 35 days. Endovascular treatment is low risk, but fails in 21% of cases, due to technical failure of access. Revascularization as a primary treatment strategy led to regression of aneurysms in 82% of cases without rehemorrhage. Direct neurosurgical excision of aneurysms is associated with a high (25% of cases) risk of complications [7]. Overall, the clinical outcome of ruptured aneurysms is poor with 41% mortality or with a severe deficit [7,8,9,10,11].

We want to demonstrate the possibilities of effective endovascular treatment navigated by simultaneous CT angiography, digital subtraction angiography (DSA), and flat panel CT on two case reports of adult women with MMD with intracranial hemorrhage from ruptured aneurysms on collateral vessels.

## 2. Case Reports

***Case 1:***

**Figure 1:**
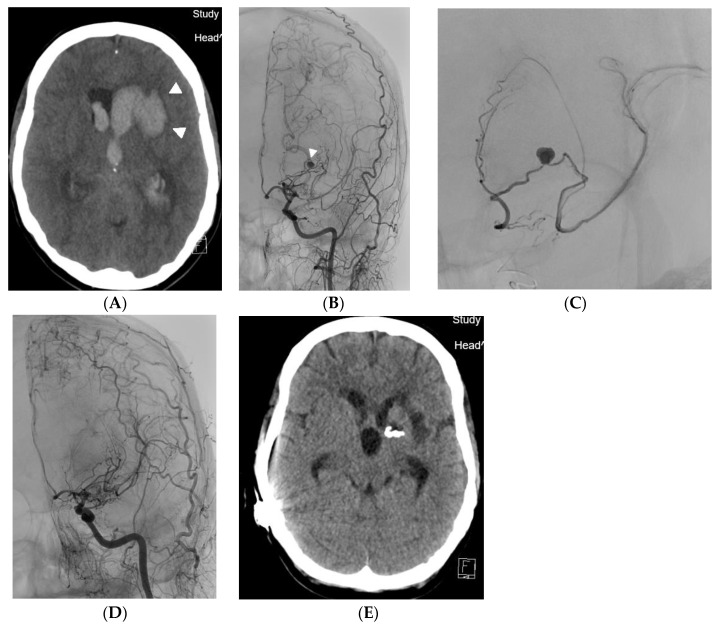
(**A**) Case 1. Native CT scan showing an intraparenchymal hematoma (pair of arrowheads) that has spread into the lateral ventricle. (**B**) Case 1. The hemorrhage was caused by an aneurysm on the lenticulostriate collateral (arrowhead), which bridges a narrow stenosis of M1 section of the middle cerebral artery. (**C**) Case 1. Selective angiography of the lenticulostriate artery with an aneurysm filling distally the M2 branch of the middle cerebral artery. (**D**) Case 1. Final angiogram after embolisation, where the aneurysm does not fill. DSA after 10 months confirmed permanent closure of the bleeding aneurysm. (**E**) Case 1. CT scan demonstrating placement of the acrylic embolisation mixture cast.

A 45-year-old female patient was admitted to the primary stroke center with sudden onset of headache, followed by somnolence and unconsciousness. The patient had a history of frequent migraines. CT scan showed a four-ventricle hematocephalus caused by a 27 × 31 × 17 mm hematoma located in the left basal ganglia (Figure 1A). Subarachnoid hemorrhage was also present. DSA (Azurion 7 B20, Philips, Best, The Netherlands) revealed a 3 mm aneurysm on hypertrophic lenticulostriate artery bridging the M1 occlusion in unilateral moyamoya syndrome (Figure 1B). Selective catheterization and distal embolisation with acrylic glue was done (Figure 1C). A guiding catheter (Guider Softip XF, Boston Scientific, Fremont, CA, USA) was inserted via a 6F sheath into the left internal carotid artery and the lenticulostriate artery was probed with a Magic 1.2F microcatheter (Balt Extrusion, Montmorency, France) with a Hybrid 0.007 inch guidewire (Balt Extrusion, Montmorency, France). Embolisation was performed with a mixture of Histoacryl (B. Braun, Melsungen, Germany) and Lipiodol Ultrafluide (Guerbet, Villepinte, France) in a 1:9 ratio to achieve good penetration of the embolising agent to the aneurysm. After embolisation, the patient received a ventriculo-peritoneal drainage for hematocephalus. Follow-up angiogram 10 months later confirmed closure of the bleeding aneurysm and showed no other aneurysm. The patient was without motoric deficit but with significant cognitive deficit required everyday assistance after her one year follow up. She confused memories with reality. Occasionally, she was given an independent task and performed it unreliably (e.g., to make coffee but bring two teas). The patient engaged in conversation at times. She was uncertain, did not remember many of the events her sister told her about (e.g., that she used to ride her bike, what her day looks like, who goes shopping), turns to her sister for advice, often cannot complete a thought. She would probably be interested in cognitive training. She would like to be more self-sufficient. So far she has had a family member with her all the time, and has not done anything on her own.

***Case 2:***

**Figure 2:**
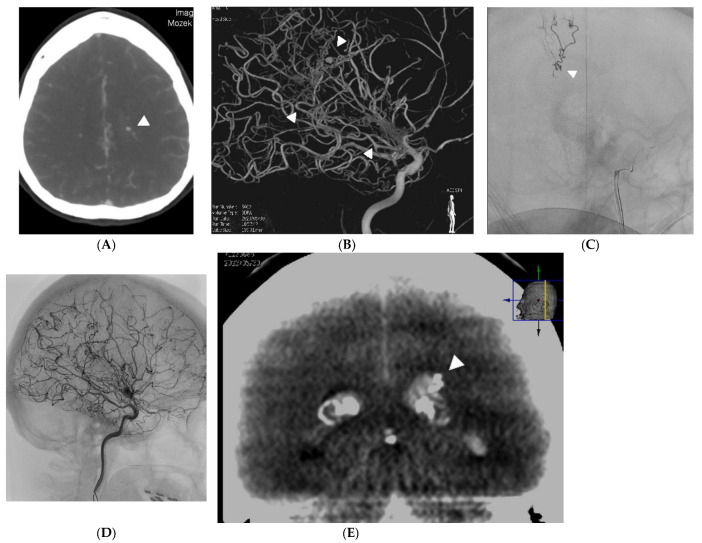
(**A**) Case 2. CT angiogram showing an aneurysm (arrowhead) in the left lateral ventricle on moyamoya collateral, which was cause of hematocephalus. (**B**) Case 2. Three dimentional angiogram showing the hypertrophic anterior chorioidal artery (pair of arrows) as collateral bridging the left internal carotid artery intracranially. The contralateral arrow indicates the aneurysm seen on the CT angiogram. (**C**) Case 2. According to the 3D angiogram, the microcatheter was navigated through the anterior chorioidal artery to the vicinity of the aneurysm. The blood flow in the artery is slowed, so the aneurysm fills only partially and the contrast agent forms a level (arrow). (**D**) Case 2. Angiogram after acrylic and oily contrast mixture injection shows that the target aneurysm is no longer filling, as well as on control angiography 2 months later. (**E**) Case 2. Flat panel CT demonstrates embolic mixture penetration into the embolised aneurysm, which was not visible during injection of the embolising agent or on angiography immediately after embolisation. Symmetric calcifications within the lateral ventricles are in the chorioid plexus.

A 47-year-old woman was admitted for a sudden onset of severe headache, CT scan showed four-ventricle hematocephalus. CTA showed a spot sign in the left lateral ventricle (Figure 2A). The patient was taken to the angiography suite and placed under general endotracheal anesthesia. A 4 mm aneurysm on the collateral vessel–anterior choroidal artery bridging the closure of the terminal segment of the internal carotid artery was diagnosed as the source of bleeding (Figure 2B). Selective catheterization of the anterior chorioidal artery using the 1.2F Magic microcatheter with 0.007 microwire, and distal embolisation with acrylic glue mixed with Lipiodol in a ratio 1:9 was done (Figure 2C). Since glue cast has not been seen on fluoroscopy and DSA, flat panel CT was used to verify penetration of glue mixture into the aneurysm (Figure 2D). After embolisation, the patient received a ventricular drainage for massive hematocephalus. The patient is without deficit and works as a teacher. The patient had a follow-up DSA two months after embolisation, which confirmed closure of the embolised aneurysm and excluded new ones. Five months later, an unsuccessful attempt was made to perform EC-IC bypass to prevent the development of another aneurysm [8]. The patient is uneventful and has been genetically proven to have MMD, which was already confirmed in her daughter before the bleeding occurred.

## 3. Discussion

Our two case reports of European women with intracranial artery occlusions demonstrate effective endovascular treatment to prevent further bleeding. The clinical outcome was determined by the presence of an intracerebral hematoma in the first patient, who is without motor deficit but with significant cognitive deficit leading to her daily dependence on the assistance of another person.

Once a clear source of bleeding is detected on CT angiography or DSA, a widely accepted strategy is to treat the lesion to prevent rebleeding. Cerebral artery aneurysms in the context of blood distribution are clear signs of the source of bleeding. In patients with MMD, a distinction is made between aneurysms of the large cerebral arteries, which are saccular and are treated with the usual endovascular or neurosurgical reconstructive methods, and aneurysms located elsewhere. In this case, they were on the distal choroidal artery, and on other moyamoya collaterals and anastomoses, including transdural ones [12]. These are preferably treated endovascularly using deconstructive methods and 3D DSA, 3D road map, and flat panel CT are used to navigate the microcatheter. These modalities, including a double-projection angiographic line, are among the standards of equipment in the neurointerventional catheterization suite. Similarly, the microcatheters we used (Magic 1.2F, Balt Extrusion, Montmorency, France) were chosen to maximize the chance of getting as close to the bleeding aneurysm as possible. The main objective was to reliably occlude the bleeding aneurysm and to minimize the surrounding ischemia in the high-eloquent area of the deep-seated hemispheric structures. The calibre of these arteries is not large and, moreover, it narrows towards the periphery. It is essential for free flow embolisation that the microcatheter does not slow or even stop the flow in the target artery, either by its size or by vasospasm due to irritation of the artery wall. Therefore, the microcatheter in this anatomical setting must be as thin and soft as possible due to its stiffness. The chosen embolisation agent ensured a permanent closure; furthermore, the microcatheter injection technique can achieve sufficient penetration to the bleeding site by dilution with an oil contrast agent and in combination with flow in the feeding artery [12,13,14,15]. In the second case, we did not detect the spread of the embolising agent, but it was evident on the control CT using a flat panel immediately after embolisation.

A literature search of aneurysm treatment in 275 MMD patients, of whom only 4.7% were Caucasian, included both large cerebral artery aneurysms and collateral aneurysms. Aneurysms on collaterals were 33.7% of the total, the other aneurysms were on circle of Willis [13]. The absence of clinical deficits in MMD was indicative of a preference for endovascular treatment over surgical treatment, particularly in aneurysms on moya moya collaterals, where 95% of patients with no or minimal deficits were free of aneurysms after endovascular treatment compared with 69% after surgical treatment [16].

Aneurysms on collaterals in MMD quite often disappear spontaneously (in 65.7% of cases) with conservative treatment [17]. Furthermore, it has been shown that revascularization surgery can lead to involution of these aneurysms and reduce the risk of bleeding [18]. A randomized trial of revascularization versus conservative therapy conducted in Japan demonstrated a reduced rate of rebleeding during follow-up in patients with hemorrhagic MMD [19]. This benefit was present in patients with aneurysms located in the posterior circulation, but not in patients with aneurysms located in the anterior circulation.

As with neurosurgical revascularization, the limitation of endovascular treatment is its availability only in specialized centers. Based on our experience and analysis of much larger sets of treatments for patients with hemorrhagic MMD, both of these treatments are effective and will continue to improve with better location of the bleeding pathology and so with more precise endovascular navigation.

In both presenting patients, the hemorrhagic event was the first sign of MMD. More specific delineation of MMD symptoms would help to make the diagnosis earlier and distinguish it from other strokes. Symptoms that could lead to a diagnosis of MMD include: visual field defects, dizziness, migraines, epilepsy, insomnia, especially if they are present in employed women, of Asian origin, with obesity, diabetes and hypertension [20].

## 4. Conclusions

Selective embolisation of ruptured aneurysms on moya moya collaterals is a simple, effective, and safe procedure when relevant microcatheters are used with imaging software navigation such as 3D DSA, 3D road map and flat-panel CT (Azurion 7 B20, Philips, Best, The Netherlands).

## Data Availability

Data are contained within the article.
